# Editorial: Real-world experience with CFTR modulator therapy

**DOI:** 10.3389/fphar.2023.1331829

**Published:** 2023-11-14

**Authors:** Burkhard Tümmler, Pierre-Régis Burgel

**Affiliations:** ^1^ Department for Pediatric Pneumology, Allergology and Neonatology, Hannover Medical School, Hannover, Germany; ^2^ Biomedical Research in Endstage and Obstructive Lung Disease Hannover (BREATH), Member of the German Center for Lung Research (DZL), Hannover, Germany; ^3^ Université Paris Cité, Institut Cochin, Inserm U1016, Paris, France; ^4^ Cochin Hospital, Assistance Publique Hôpitaux de Paris, Paris, France

**Keywords:** elexacaftor, tezacaftor, ivacaftor, CFTR, cystic fibrosis

Cystic fibrosis (CF) is a severe autosomal recessive trait that is caused by mutations in the *Cystic Fibrosis Transmembrane Conductance Regulator* (*CFTR*) gene. The basic defect impairs the transport of chloride and bicarbonate across the apical membrane of epithelial cells leading to mucus plugging of the ducts of exocrine glands. Mucus obstruction of the small conducting airways predisposes to a vicious cycle of infection, inflammation and airway remodeling, which determine the course and prognosis of most people with CF (pwCF). Being a fatal disease with death in early childhood in the 1950s, the symptom-oriented treatment programs have gradually improved the median life expectancy in countries with well-established CF care to about 50 years in 2020.

The *CFTR* gene was identified in 1989. By the mid-1990s high-throughput screening programs were initiated to identify small molecules that improve the function of mutant CFTR. The first molecule approved for use in humans is the CFTR potentiator ivacaftor (IVA), which facilitates the opening of the CFTR ion channel. In 2010 IVA monotherapy became available for the small group of pwCF who are harboring a gating mutation in the *CFTR* gene. Of the more than 2,000 known disease-causing mutations, the 3-base pair deletion of codon 508, named p.Phe508del, is the most common mutation present in 50%–90% of CF alleles in European populations. p.Phe508del CFTR is primarily defective in folding and processing of the nascent protein at the endoplasmic reticulum. CFTR correctors stabilize critical sites during folding. The first approved correctors, lumacaftor (LUM) and tezacaftor (TEZ), in combination with IVA showed only modest improvements of lung function in phase 3 clinical trials, but they reduced the frequency of pulmonary exacerbations and have been predicted to increase the median survival by 7 years compared to symptom-oriented care (Lopez et al., 2023).

By November 2019 the outcome of phase 3 studies of the triple therapy with elexacaftor (ELX), TEZ and IVA in CF patients with one or two p.Phe508del alleles were published (Heijerman et al., 2019; Middleton et al., 2019). Study participants substantially improved in lung function, body weight and quality of life measures classified as a game changer of CF disease. Hence, the projected survival for pwCF treated with ELX/TEZ/IVA has been predicted to show large improvements in future years (Lopez et al., 2023). Consistent with this estimate, the reconstituted ELX/TEZ/IVA - Phe508del CFTR complex showed wild type conformation in cryoelectron micrographs (Fiedorczuk and Chen, 2022).

The 15 contributions of this Research Topic report on the “*Real-world experience with CFTR modulator therapy*.” One of us (Tümmler) summarized the take-home message of all post-approval studies on ELX/TEZ/IVA that were published until February 2023.

Stanke et al. compared the intestinal Phe508del CFTR glycoforms at baseline and during ELX/TEZ/IVA therapy. By applying a novel protocol to separate CFTR protein isoforms with high resolution, complex glycosylated Phe508del CFTR was found to be less glycosylated and less polydisperse than mature wild type CFTR both in absence and presence of ELX/TEZ/IVA indicating that triple therapy rescues Phe508del CFTR at the endoplasmic reticulum, but does not normalize post-translational processing at the Golgi apparatus.

The FDA approved ELX/TEZ/IVA for treatment of pwCF who are carrying *CFTR* mutations that are responsive to modulator *in vitro* or *in vivo*. Carriage of two loss-of-function mutations does not fulfill the label. However, as shown by Pallenberg et al. for two *prima facie* loss-of-function splice mutations, ELX/TEZ/IVA conferred residual CFTR activity to some, but not all examined organs. This showcase tells us that the individual response of rare CFTR mutations to highly-effective CFTR modulation cannot be predicted from assays in standard cell cultures, but requires the personalized multi-organ assessment by CFTR biomarkers.

ELX/TEZ/IVA improved Phe508del CFTR function in intestinal epithelia to a level of about 40% of normal in pwCF aged 12 years and older (Graeber et al., 2022a). Berges et al. now shows that the clinically less effective combination therapy with LUM/IVA improved Phe508del CFTR activity in the intestine to about 30% of normal in children aged 2–11 years exceeding the 18% of normal found previously in pwCF aged 12 years and older. Consistent with registry reports on ivacaftor, CFTR modulator therapy is more efficient in the young age group to attenuate the basic defect than in the elderly suggesting that with early initiation of treatment the long-term outcome will be better.

The first phase 3 clinical trials on triple therapy assessed pwCF aged 12 years and older and a lung function of ppFEV1 of 40%–90% predicted of normal. The real-world studies now tell us that pwCF with severe airway obstruction (ppFEV_1_ <40) and pwCF with well-preserved lung function (ppFEV_1_ >90) showed less improvement in ppFEV1 than pwCF with ppFEV1 40–90 (Fila et al.). The clinical response towards ELX/TEZ/IVA depends on age and lung disease severity. Adolescents and children showed benefit in nutritional status and pulmonary function (Olivier et al.), especially the individuals with more severe lung disease prior to ELX/TEZ/IVA (within the lowest 25% of age specific ppFEV1) showed higher improvements in spirometry compared to adults in this severity group (Schütz et al.). Improvements in FEV1 upon initiation of ELX/TEZ/IVA therapy were demonstrated to go hand in hand with a lower bacterial load and less frequent detection of *Aspergillus fumigatus* in respiratory secretions (Eschenhagen et al.), a decrease of ventilation inhomogeneity and a reduction of structural lung damage (Graeber et al., 2022b; Appelt et al.). Likewise, paranasal sinus abnormalities, particularly mucopyoceles, stably decreased during treatment with LUM/IVA in p.Phe508del homozygous children with CF (Wucherpfennig et al.).

Highly effective CFTR modulation does not only substantially improve lung function, but typically also immediately improves pH and fluid homeostasis in the intestine. Nutrients are more efficiently absorbed leading to an increase of the body mass index. When ELX/TEZ/IVA therapy is initiated, abdominal symptoms may transiently emerge. PwCF often report more flatulence and abdominal pain during the first 10 days (Mainz et al.), but these abdominal symptoms improve or even disappear within the following 2 weeks.

An increase of the body mass index has been interpreted in the phase 3 CFTR modulator trials as an improvement of anthropometry. The CF clinic in Toronto now shares their experience with us that the gain of body weight is mainly due to an increase of fat mass whereas the lean body mass does not improve (Mouzaki et al.). If we want to avoid to see many obese pwCF in the future, pwCF should rapidly switch from a calorie-rich diet recommended in the pre-modulator era to the balanced mixed diet of the healthy population.

The phase 3 trials and the open-extension study did not find any neurologic or psychiatric side effects of ELX/TEZ/IVA therapy other than headache. Post approval, however, substantial mental health side effects have been reported, which impacted on day-to-day activity and quality of life (Spoletini et al., 2022). Ibrahim et al. from the CF clinic in Cork now share their experience with a dose reduction strategy in CF adults who developed anxiety, irritability, sleep disturbance and/or mental slowness after initiation of full dose treatment with ELX/TEZ/IVA. Initial discontinuation or reduction of medication and subsequent dose escalation every 4–6 weeks resulted in resolution of mental/psychological adverse events, without loss of clinical effectiveness. To put the numerous case reports on mental status changes into perspective, the CF clinic at the Charité in Berlin performed the worldwide first prospective study on the relationship between initiation of ELX/TEZ/IVA therapy and changes in mental health in CF adults (Piehler et al.). Symptoms of depression decreased, but symptoms of anxiety did not change after 8–16 weeks of treatment with ELX/TEZ/IVA. Hence, the rapid attenuation of the symptoms of CF disease upon initiation of ELX/TEZ/IVA will improve the quality of life and depressive symptoms in most pwCF, but 5%–10% of CF adults experience adverse events of mental health that deserve particular attention in the future.

CFTR modulator drugs are expensive. ELX/TEZ/IVA is not available in all countries around the globe, particularly pwCF living in low-middle income countries have currently no access to ELX/TEZ/IVA. Zampoli et al. discuss in their minireview the global inequality in the access to the life-altering CFTR modulator drugs. The CF community is asked to deal with this real-world disparity.

Finally, CFTR modulators are variant-selective therapies. Although ELX/TEZ/IVA has been primarily developed for patients with at least one p.Phe508del mutation, it has become clear that many other mutations respond to ELX/TEZ/IVA (Burgel et al., 2023). Determining which mutations respond to ELX/TEZ/IVA has the potential of improving health status and survival in responders. For those who do not respond, newer therapeutic options should be developed.
